# Role of the Epigenetic Regulator HP1γ in the Control of Embryonic Stem Cell Properties

**DOI:** 10.1371/journal.pone.0015507

**Published:** 2010-11-15

**Authors:** Maïa Caillier, Sandrine Thénot, Violaine Tribollet, Anne-Marie Birot, Jacques Samarut, Anne Mey

**Affiliations:** Institut de Génomique Fonctionnelle de Lyon, Université de Lyon, Université Lyon 1, CNRS, INRA, Ecole Normale Supérieure de Lyon, France; National Institute on Aging (NIA), National Institutes of Health (NIH), United States of America

## Abstract

The unique properties of embryonic stem cells (ESC) rely on long-lasting self-renewal and their ability to switch in all adult cell type programs. Recent advances have shown that regulations at the chromatin level sustain both ESC properties along with transcription factors. We have focused our interest on the epigenetic modulator HP1γ (Heterochromatin Protein 1, isoform γ) that binds histones H3 methylated at lysine 9 (meH3K9) and is highly plastic in its distribution and association with the transcriptional regulation of specific genes during cell fate transitions. These characteristics of HP1γ make it a good candidate to sustain the ESC flexibility required for rapid program changes during differentiation. Using RNA interference, we describe the functional role of HP1γ in mouse ESC. The analysis of HP1γ deprived cells in proliferative and in various differentiating conditions was performed combining functional assays with molecular approaches (RT-qPCR, microarray). We show that HP1γ deprivation slows down the cell cycle of ESC and decreases their resistance to differentiating conditions, rendering the cells poised to differentiate. In addition, HP1γ depletion hampers the differentiation to the endoderm as compared with the differentiation to the neurectoderm or the mesoderm. Altogether, our results reveal the role of HP1γ in ESC self-renewal and in the balance between the pluripotent and the differentiation programs.

## Introduction

Embryonic stem cells (ESC) are the pluripotent cells that give rise to the differentiated cells of the three germ layers at the earliest stages of development (endoderm, mesoderm and ectoderm) [1]. In mice, these cells are derived from the inner cell mass of blastocysts and are capable of prolonged self-renewal in vitro. ESC maintenance is supported by a conserved and restricted set of key transcription factors, namely Oct4 [2], [3], Nanog [4], [5] and Sox2 [6], [7].

Characterization of these cells has shown that the progression from a pluripotent to differentiated status is correlated with chromatin condensation [8] and enrichment in silenced chromatin marks (see [9] for review) through heterochromatin formation. In contrast, the chromatin in ESC is relaxed with loosely attached architectural proteins [10] and a globally permissive transcriptional state [11] characteristic of euchromatin. Recently, RNA interference screens targeting chromatin-associated proteins have revealed the existence of ESC regulations at the chromatin level that contribute to their open chromatin state and that are important for ESC properties, namely their self-renewal and their pluripotency [12], [13].

It has also been shown that epigenetic modifiers involved in the methylation status of H3K9 contribute to ESC maintenance under the control of the transcription factor Oct4 [14], [15]. This mark is involved in heterochromatin formation and in the permanent silencing of specific genes in transcriptionally active euchromatic regions when appropriate [16], [17]. Notably the ESC-specific H3K9 methyltransferase ESET is involved in pluripotency maintenance through the repression of differentiation genes [15]. The inducible H3K9 methylation also contributes to the epigenetic flexibility associated with the commitment to differentiation, as revealed by the requirement for the euchromatin-associated G9a H3K9 methyltransferase during early embryonic development [18] as well as in the silencing of euchromatic loci during ESC differentiation [19]. Methylated H3K9 marks are poorly represented in ESC when compared with marks associated with active genes [20], [21] and it is not known whether epigenetic regulators recognizing methylated H3K9 sustain ESC identity with similar or distinct functions when compared with other regulators on activation marks [12], [13].

Methylated H3K9 marks are recognized by epigenetic regulators in the heterochromatin proteins 1 (HP1) family, which is conserved in a large number of species [22]. Mammals have three HP1 variants: α, β and γ. These proteins are capable of homo- or heterodimerizing and recruiting large protein complexes that are involved in gene regulation (see [23] for review). Despite their structural resemblance to one another, the three isoforms have some distinct, non-redundant functions [24] and localization patterns. HP1γ, and to a lesser extent HP1β, localizes not only to heterochromatic sites, as does HP1α [25] but also to euchromatic region [26] to repress gene transcription. Localization of HP1 proteins is submitted to important changes during differentiation [27]–[28] which probably accounts for the chromatin organization during ESC differentiation. However, HP1γ is also associated with the transcriptional activation of direct target genes [29]–[32]. This situation suggests that, unlike other HP1 isoforms, HP1γ may sustain gene expression in ESC. This characteristic reconciliates repressive chromatin marks with transcriptional activity and may provide flexibility for the rapid reprogramming that occurs during ESC differentiation.

To test this hypothesis, we have explored the functional consequences of HP1γ deprivation on mouse ESC maintenance and differentiation. We show in this study that HP1γ regulates ESC identity being involved in ESC self-renewal and in the balance between pluripotency and differentiation.

## Results

### Embryonic stem cells with low levels of HP1γ show reduced self-renewal efficiency

**Figure 1:**
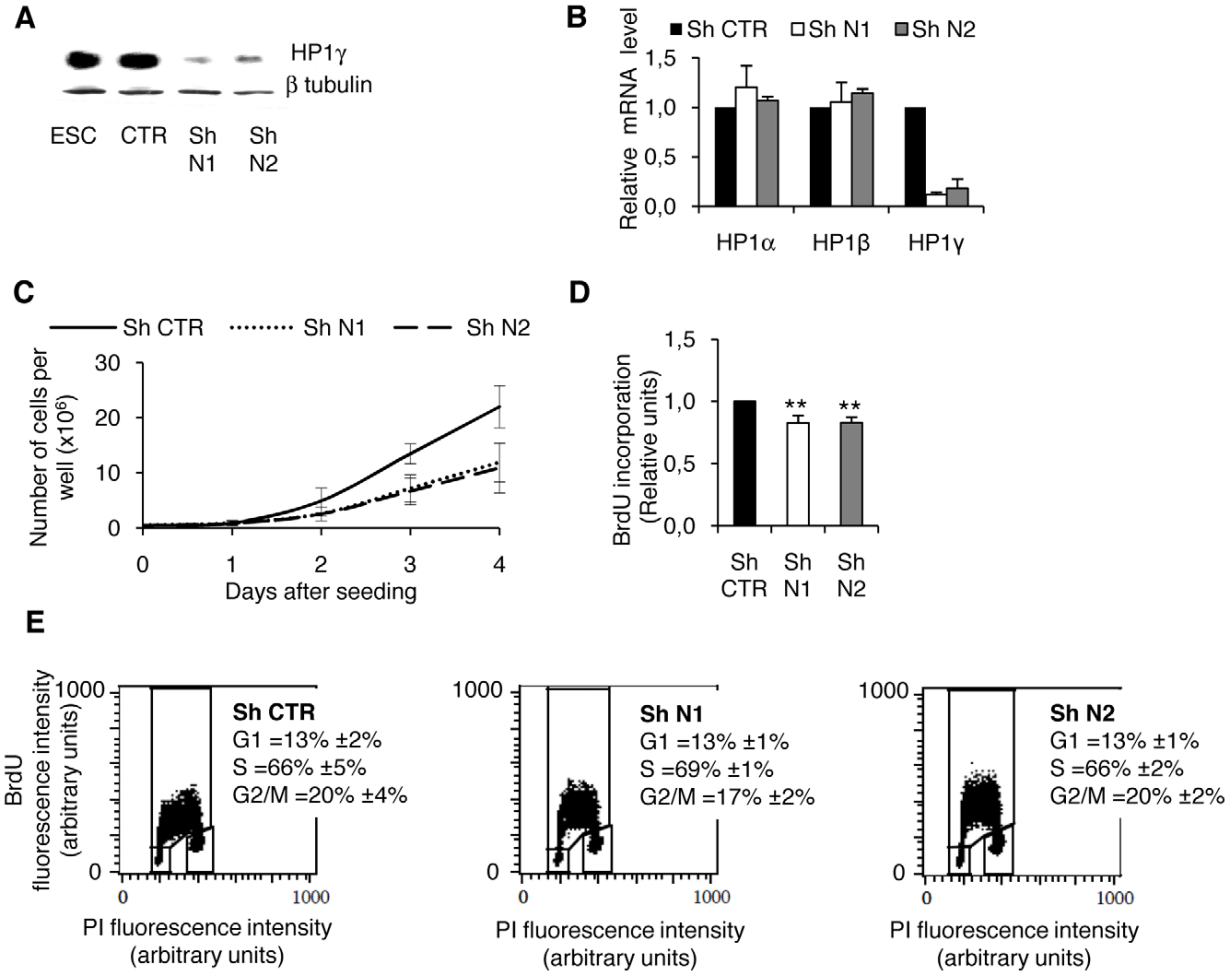
HP1γ knockdown ESC exhibit an altered proliferation rate. A. Two different shRNA directed against HP1γ (shN1 and shN2) were compared to a control shRNA (shCTR). Knockdown efficiency was measured by western blot using an anti-HP1γ antibody and compared to the basal level expressed in untransduced ESC and in shCTR cells. Beta-tubulin was used as a protein loading control. B. mRNA levels of the three HP1 isoforms HP1α, β and γ were measured by RT-qPCR. Values are represented relative to the ones obtained from shCTR-transduced cells. C. Growth curves for the shCTR, shN1 and shN2 cell lines representing the number of cells obtained after 1, 2, 3 or 4 days of culture. D. Proliferation measured by BrdU incorporation. BrdU was added to exponentially proliferating cells during the last 8 hours of culture. Results are represented relative to the value obtained in the shCTR cell line. Results are the means of three independent experiments. ** p<0,002 by t test. E. Cell cycle analysis using propidium iodide (PI) and BrdU incorporation. All values are means +/− SD from three independent experiments.

The role of HP1γ in ESC was examined by RNA interference via lentiviral vectors encoding a short hairpin RNA (shRNA). Four shRNA constructs designed against HP1γ were tested in comparison to a control construct (shCTR) with at least 4 base pair mismatches to any known mouse gene. Among the four shRNA constructs tested, two (shN1 and shN2) specifically decreased the level of HP1γ mRNA by more than 80% and were used in subsequent experiments. Western blot analysis showed that after 6 days of puromycin selection to maintain the shRNA construct, the level of HP1γ protein was dramatically decreased in HP1γ shN1 and shN2 cell lines compared to shCTR cells (Figure 1A). The N1 and N2 shRNA did not induce any decrease in the levels of HP1α and HP1β mRNAs, demonstrating their specificity toward HP1γ (Figure 1B).

In the presence of puromycin, shHP1γ ESC could be maintained in culture and showed no evidence of morphological change (not shown). However, both lines grew more slowly than did control ES cells. To quantify this proliferative defect, cells were plated at low density and counted daily. The growth curves obtained (Figure 1C) demonstrated that the cell growth was reproducibly reduced in both shHP1γ cell lines compared to control ESC. This observation was confirmed by a proliferation assay that measured BrdU (BromodeoxyUridine) incorporation in newly synthesized DNA during a unique cell cycle and which showed around 20% decrease in DNA synthesis in the shHP1γ cell lines compared to the shCTR line (Figure 1D).

To explore the underlying cause of this proliferation defect, we investigated cell death by measuring the incorporation of the nucleic acid intercalating agent propidium iodide (PI) which is actively excluded from viable cells and retained in membrane-permeant dead cells. To detect early cell death events, annexin V labeling was used to detect the translocation of this phosphatidylserine protein from the inner to the outer leaflet of the plasma membrane occurring only in apoptosing cells [33]. The mean percentage of total dead cells was quite similar in shHP1γ cells and in shCTR cells (CTR : 6±2%, N1: 7±2%, N2 = 7±2%, measured from three independent experiments), and therefore could not explain the cell number decrease specifically observed upon HP1γ knockdown. Another explanation for the lower growth of cell population could be cell cycle arrest. We performed cell cycle analyses using propidium iodide and BrdU to label proliferating cells (Figure 1E). The cell cycle distribution of the cells was not affected by HP1γ repression.

Altogether, these results demonstrate that the down regulation of HP1γ in mESC leads to a decrease in cell growth that is not the consequence of blockage in the cell cycle or of an increase in cell death. It thus appears that the cell growth decrease observed in HP1γ depleted cells is due to an overall slowing down of the cell cycle. To better understand the mechanisms of the proliferation defect in shHP1γ cells, a microarray analysis of their transcripts was undertaken and compared with those in control cells. As shown in Table 1, 34 genes were differentially regulated between the two cell lines with a fold change higher than 2.2. Very strikingly, 6 of the 18 genes with a known function are associated with the positive regulation of cell growth. All of these 6 genes were down regulated (variations confirmed by RT-qPCR, Table S1), while one gene associated with the repression of cell proliferation was induced. Hence, these results show that HP1γ is involved in the regulation of ESC self-renewal most probably through the direct or indirect control of cell growth-associated genes.

**Table 1:** Genes misregulated following HP1γ knock-down in proliferating ESC.

Gene symbole	Gene Name^a^	Fold change	Fonction^b^
Mep1b	Meprin 1 beta	5.3	Tissue repair ; Cell migration; Modulation of the immune system
Frmd4b	FERM domain containing 4B	5.2	Unknown
Aass	Aminoadipate-semialdehyde synthase	4.4	Lysine degradation pathway
Hspb7	Heat shock protein family, member 7	4.2	Unknown
AI662270	Expressed sequence AI662270	3.9	Unknown
Fv1	Friend virus susceptibility 1	3.6	Inhibition retroviral infection
Mthfd2l	Methylenetetrahydrofolate dehydrogenase2- like	3.5	Unknown
Clca4	Chloride channel calcium activated 4	3.3	Chloride transport
* Ifitm3	Interferon induced transmembrane protein 3	3.1	Repression of cell proliferation; Cell adhesion
2310043M15Rik	RIKEN cDNA 2310043M15 gene	3.0	Unknown
Il1rl2	Interleukin 1 receptor-like 2	2.6	Unknown
Ap1s3	Adaptor-related protein complex AP-1,sigma 3	2.6	Cargo protein
Gtsf1	Gametocyte specific factor 1	2.5	Unknown
1200003I07Rik	RIKEN cDNA 1200003I07 gene	2.4	Unknown
D4Wsu114e	Migration and invasion inhibitory protein	2.2	Inhibition of cell invasion and migration
Hydin	Hydrocephalus inducing	−11.2	Cilia Motility
Msln	Mesothelin	−5.6	Unknown; Associated with ovarian cancers and mesotheliomas
Cbx3	Chromobox homolog 3 ( HP1g)	−4.9	Cbx3
Taok3	TAO kinase 3	−4.0	Unknown
* Aldh3a1	Aldehyde dehydrogenase family 3,subfamily A1	−4.0	Activation of cell growth
* Gpx2	Glutathione peroxidase 2	−3.8	Activation of cell growth of cancer cells; Inhibition of migration and invasion; Regulation of hyperoxides level
* Pla2g1b	Phospholipase A2, group IB	−3.6	Activation of cell growth ; Cell migration; digestion of glycerophospholipids
Cabp4	Calcium binding protein 4	−3.5	Important for normal synaptic function
LOC331480	Predicted gene, EG331480	−3.4	Unknown
Tmem40	Transmembrane protein 40	−3.4	Unknown
Tmprss5	Transmembrane protease serine 5 (spinesin)	−3.2	Unknown
Acoxl	Acyl-Coenzyme A oxidase-like	−3.1	Unknown
Myom2	Myomesin 2	−3.0	Interconection of the major structure of sarcomeres
Crxos1	Crx opposite strand transcript 1	−3.0	Unknown
* Ckmt1	Creatine kinase mitochondrial 1	−3.0	Activation of cell growth ; Cell viability; Cellular energy homostasis
Fxyd4	FXYD domain-containing ion transportregulator 4	−2.9	Unknown
Ggt1	Gamma-glutamyltransferase	−2.9	Glutathione metabolism ; Regulation of osteoclast biology
*Calml4	Calmodulin-like 4	−2.8	Activation of cell growth
*Nrp2	Neuropilin 2	−2.2	Activation of cell growth; Angiogenesis; Migration of cancer cells; Functions in nervous system

a Genes with a fold change higher than 2.2 in three independent chip hybridization experiments;

b Functions given based on bibliography analysis.

*Genes affecting cell proliferation.

### The knock-down of HP1γ increases the propensity of ESC to differentiate

**Figure 2:**
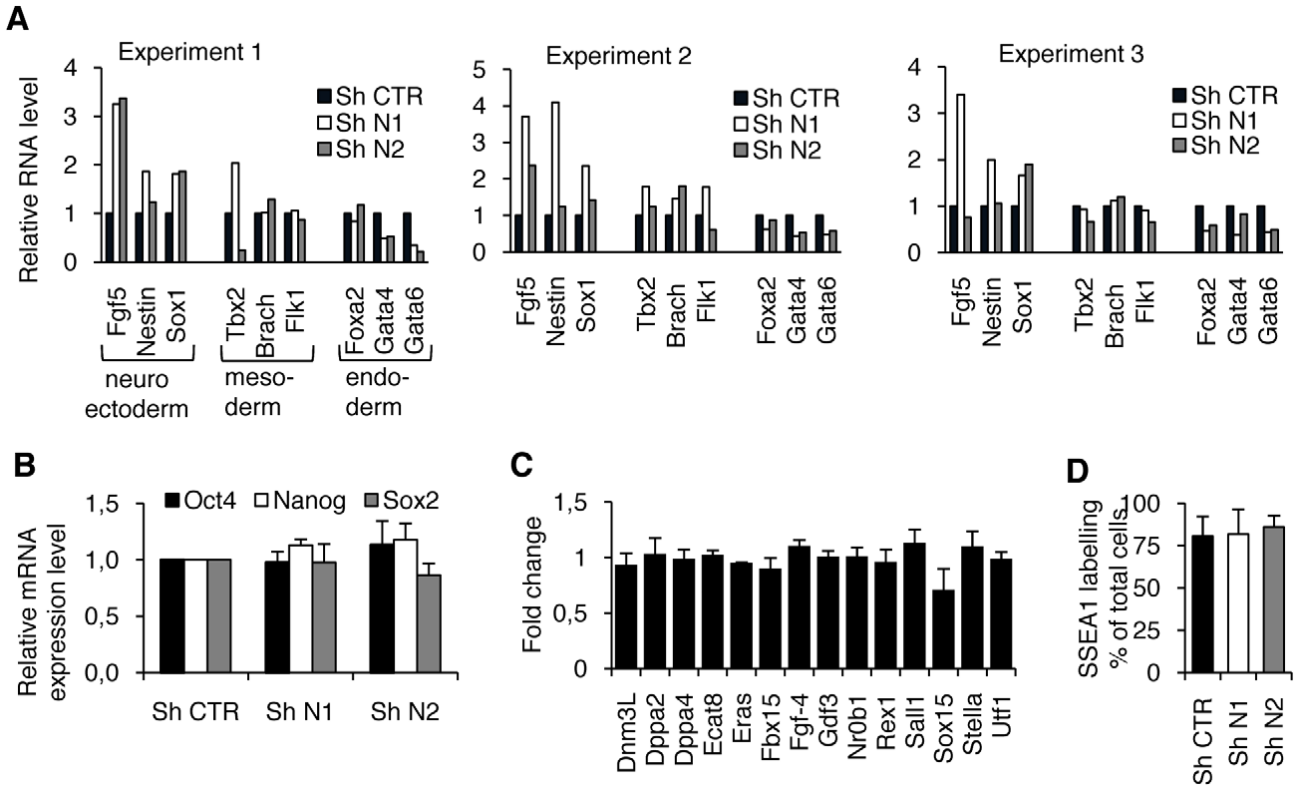
HP1γ knocked-down ESC sporadically express differentiation markers but show normal expression of pluripotency markers. A. The expression of the indicated genes representing the three germ layers (neuroectoderm, mesoderm and endoderm) was measured by RT-qPCR. Results from three independent RNA samples are reported to illustrate the variability of the results obtained when genes are analyzed individually. B. The RNA expression levels of the core set of transcription factors required to sustain pluripotency was measured in shRNA cell lines by real time PCR. C. Fold change of additional markers associated with pluripotency obtained from microarrays analysis. Results are mean +/− SD of three independent microarrays experiments. D. The proportion of undifferentiated cells in the three shRNA cell lines was measured by immunolabelling of SSEA1 and subsequent flow cytometry analysis. In A, B and C results are reported as a ratio of the values obtained in the shCTR cells. For B and D, the means and standard errors were calculated from four independent experiments.

Because the slowing down of proliferation and the commitment to differentiation are known to be correlated in ESC, we wondered whether this balance was disrupted in HP1γ knockdown cells. Accordingly we explored the expression level of differentiation associated genes by RT-qPCR. The results from three independent RT-qPCR experiments indicate that shHP1γ cells sporadically expressed differentiation markers in proliferative conditions (Figure 2A). The two shHP1γ cell lines did not always exhibit the same levels of a given transcript and distinct degrees of variation were observed between the experiments in all cell lines. However, when considering all the markers of a specific germ layer, a reproducible tendency toward the induction of neuroectoderm differentiation markers (Fgf5, Nestin, and Sox1) was observed in shHP1γ cells. The inductions of some mesoderm markers were detected but with very low intensity and with a strong variability. On the contrary the basal level endoderm markers (Foxa2, Gata4, Gata6) seemed rather lowered. These results suggest that shHP1γ cells are poised to differentiate, though they have not yet specifically done so. Indeed, the expression of the pluripotency genes was maintained. As shown in Figure 2B, the mRNA expression levels of Oct4, Nanog and Sox2 were similar in both shHP1γ cells and control cells, indicating that the decrease in HP1γ expression did not affect the genes controlling ESC maintenance or pluripotency at the population level. The microarray analysis confirmed that the decrease in HP1γ protein does not affect the expression level of 13 other genes reported to be associated with pluripotency [34] (Figure 2C). Moreover, we quantified the presence of the pluripotent stem cell antigen SSEA1 (Stage-Specific Embryonic Antigen-1) by flow cytometry. The proportion of SSEA1-positive cells was similar (around 80% of the whole population) in control and in shHP1γ cells (Figure 2D). Altogether this data indicate that the same proportions of cells harboring pluripotency markers could be found in CTR, N1 and N2 populations.

**Figure 3:**
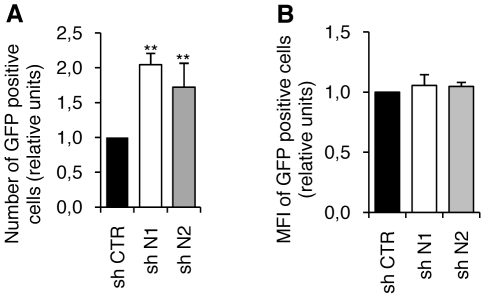
HP1γ knockdown increases the proportion of cells expressing the differentiation marker Brachyury. ESC transfected with a Brachyury promoter-GFP construct were further transfected with the three shRNA. The promoter activity was followed by GFP expression measured by flow cytometry. A. The proportion of GFP positive cells is reported as the ratio of the value obtained in the shCTR cells. B. Representation of the mean fluorescence intensities (MFI) in GFP positive cells. The means and standard errors were calculated from four independent experiments. *** p<0.02 by t test.

The increased expression of differentiation markers in HP1γ knockdown cells could reflect an increased background of gene expression in all cells, or alternatively an increase in the number of cells expressing basal levels of these markers. To distinguish between these two hypotheses ES cells were stably transfected with a construct where GFP expression was placed under the control of the promoter of the mesodermal marker brachyury [35]. Engineered ES cells were subsequently transfected with shRNA CTR, N1 or N2. The knock-down of HP1γ strongly increased the percent of brachyury GFP cells (Figure 3A) but the mean fluorescence intensity was not affected (Figure 3B). These observations confirm that mesoderm markers were also induced despite the low and sporadic increase of brachyury transcripts (Figure 2A) in the whole ES cells population. Altogether qPCR analysis and the reporter assay indicate that lowering the level of HP1γ increased the number of ES cells that are prone to differentiate toward the neurectoderm and the mesoderm.

**Figure 4:**
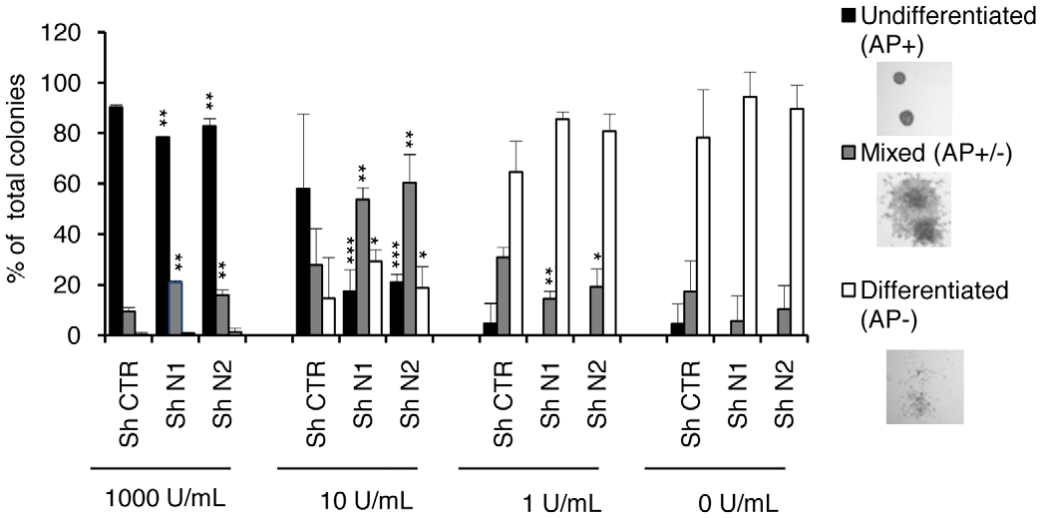
A low level of HP1γ favors ESC commitment to differentiation. The three shRNA cell lines (shN1, shN2 and shCTR) were seeded at low density and cultured with medium containing the indicated concentrations of LIF; the concentration of 1000 U/ml corresponds to that used in proliferating medium. Five days after seeding, colonies were fixed, stained for alkaline phosphatase and scored as undifferentiated (AP+), mixed (AP+/−) or differentiated (AP-). For each concentration of LIF, results are represented as the percent of the total number of colonies. Means and standard errors were calculated from three independent experiments, and t tests were performed to determine the significance of the differences between shHP1 and shCTR cells at each LIF concentration for a given type of colony (AP+, AP- or mixed). * p<0.1, ** p<0.05, *** p<0.02.

The maintenance of mouse ESC is sustained by the LIF cytokine in the culture medium [36]. To quantify this increased propensity to differentiate we performed a colony-forming assay with decreasing LIF concentrations (Figure 4). After 5 days of culture, the differentiation status of colonies was analyzed by alkaline phosphatase (AP) staining, which specifically marks pluripotent cells. The colonies were scored as undifferentiated, mixed or differentiated. The answers to LIF privation were dose-dependent but more pronounced in shHP1γ cell lines. Specifically, we noticed that seeding the cells in culture medium supplemented with only 10 U.mL^−1^ of LIF induced the formation of a significantly increased number of mixed colonies by shRNA-treated ESC compared to control cells. Indeed, in this condition the vast majority of colonies formed by N1 and N2 cells were mixed or differentiated (respectively 83% and 81%), as compared to less than 40% for CTR colonies. The same effect was also seen in the presence of 1 U.mL^−1^ with no undifferentiated and more differentiated colonies that were scored in N1 and N2 than in CTR cells.

Taken together these results show that low levels of HP1γ increase the propensity of ESC to differentiate but do not induce their spontaneous progression toward differentiation. They rather induce ESC to enter a metastable state more sensitive to differentiating conditions.

### Low levels of HP1γ increase ESC differentiation efficiency but restrict the differentiation pattern

**Figure 5:**
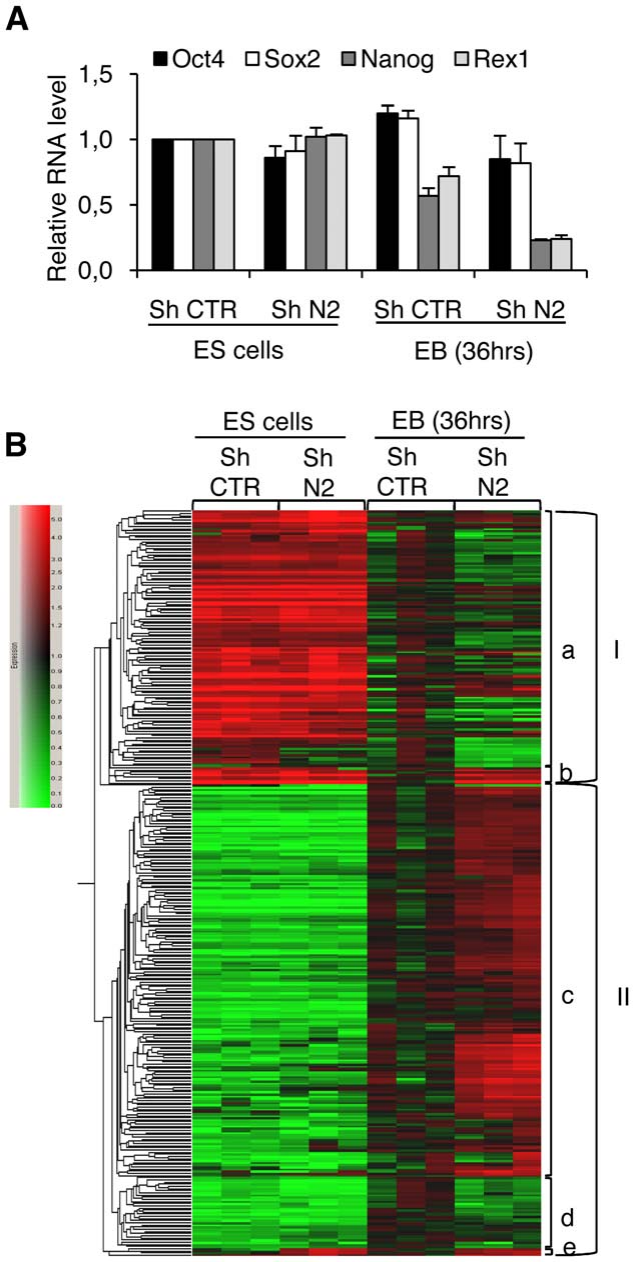
HP1γ knockdown improves early differentiation of ESC. The transcriptome of shN2 cells was compared to that of shCTR cells using Affymetrix chip hybridization. RNA were isolated from cells that were cultivated in the absence of LIF and in non-adherent conditions to form embryoid bodies (EB) over the course of 36 hours. A. RNA levels of the three pluripotent transcription factors measured by real time PCR in the same RNA extracts that were compared by microarrays analysis. B. Variation of genes associated with differentiation are represented relative to the mean value obtained in undifferentiated shCTR RNA. Upregulated expression is indicated in red, downregulated expression is indicated in green; the variations obtained from the three independent differentiation experiments are represented.

The above data suggest that HP1γ may be involved in the very early steps of differentiation, during the commitment process. An early time point of differentiation was therefore analyzed for gene expression in embryoid bodies (EB) formed over a 36 h period by shCTR and shN2 cells. At this time point, Oct4 and Sox2 were still expressed but the other core pluripotency transcription factor, Nanog, was already repressed (Figure 5A). Associated with the repression of Rex1, this indicates that the cells were committed to differentiation but that the differentiation was not fully achieved. Differentiation markers (list generated by an automatic analysis of the bibliography and displayed as supplementary material File S1) were selected for analysis and clustered based on gene expression (Figure 5B).

During differentiation the genes were either down- or upregulated (Figure 5B, groups I and II, respectively). The genes whose expression changed during differentiation globally displayed the same regulation in control cells and in HP1γ knockdown cells but with a notably higher amplitude (Figure 5B, clusters a and c). A restricted number of genes showed either impaired variation when compared with control cells (clusters b and d) or variation restricted to cells with low levels of HP1γ (Figure 5B, cluster e), but their analysis did not reveal any convergent function. These data thus indicate that the decrease of HP1γ level probably favors the differentiation of embryonic stem cells from the earliest steps by increasing the expression of differentiation markers.

**Figure 6:**
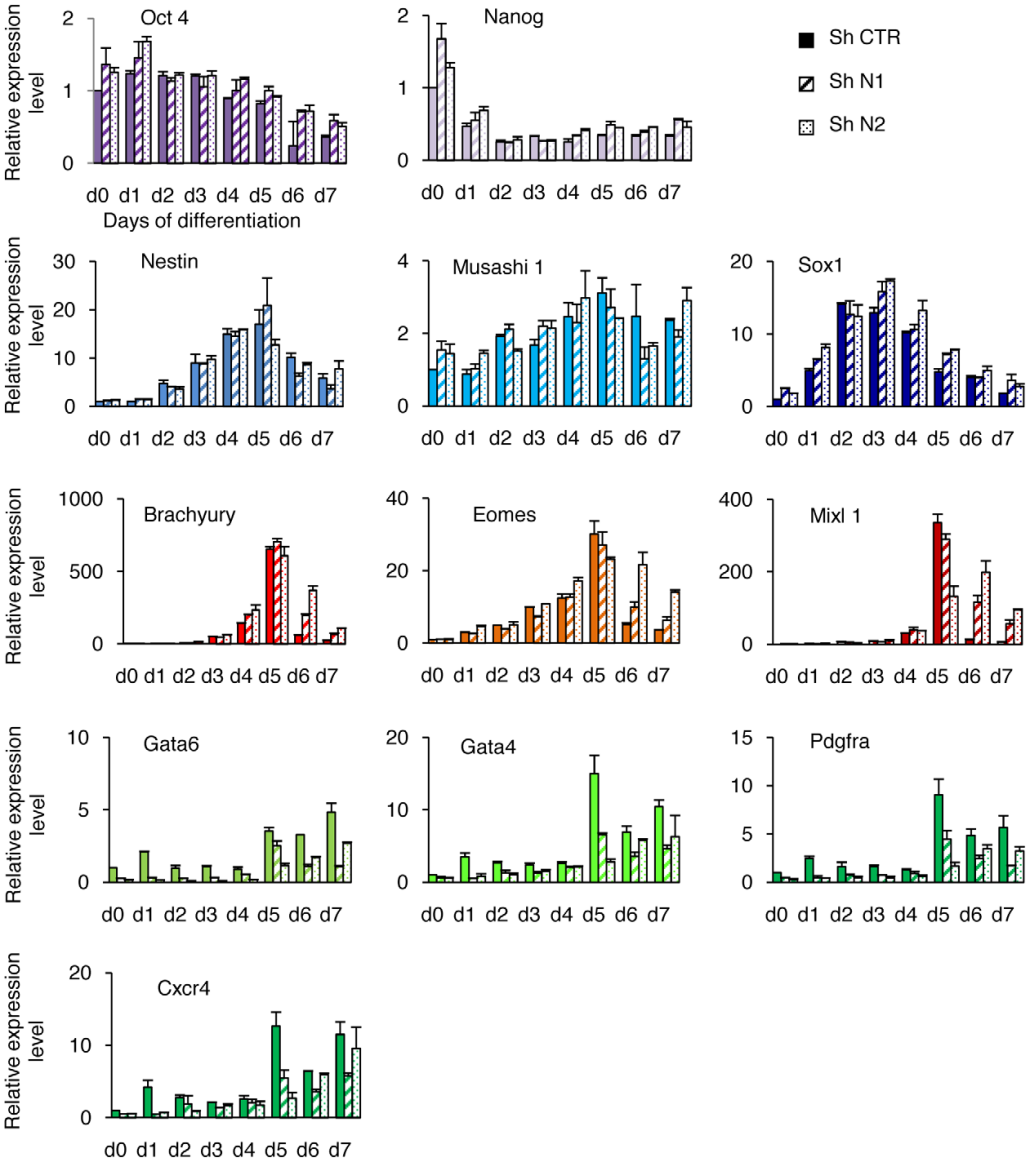
HP1γ deprivation of ESC specifies differentiation during embryoid bodies formation. The three shRNA cell lines (shN1, shN2 and shCTR) were induced to differentiate by embryoid bodies formation. Differentiation efficiency was assessed following the decrease of pluripotency markers (purple). Transcript levels of markers representing the three germ layers (neuroectoderm in blue, mesoderm in red and endoderm in green) were measured by RT-qPCR each day (d) after seeding. Results represented relatively to the value obtained in ESC (d0), are the mean of duplicates +/− SD from one experiment representative of two.

**Figure 7:**
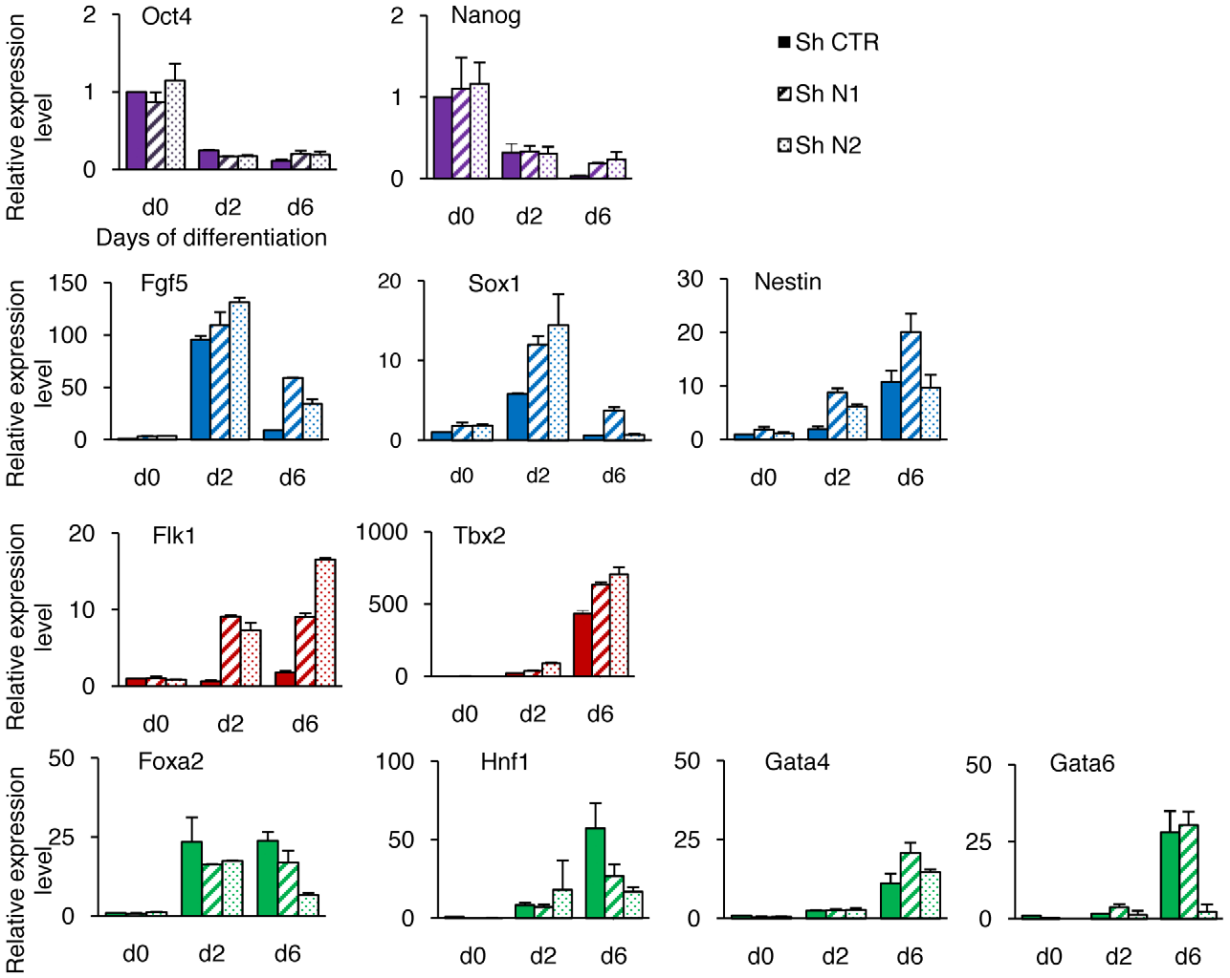
Specification of ESC differentiation by HP1γ deprivation is also observed in retinoic acid-induced cells. The three shRNA cell lines (shN1, shN2 and shCTR) were induced to differentiate using retinoic acid. Differentiation efficiency was assessed following the decrease of pluripotency markers (in purple). Transcript levels of markers representing the three germ layers (neuroectoderm in blue, mesoderm in red and endoderm in green) were measured by RT-qPCR after 2 or 6 days of differentiation. Results represented relatively to the value obtained in ESC (d0), are the mean of duplicates +/− SD from one experiment representative of three.

To determine whether HP1γ knockdown also affected the later phases of differentiation a kinetic study of gene expression in embryoid bodies cultivated for 1 to 7 days was done by RT-QPCR (Figure 6). The down-regulation of the pluripotency genes Oct4 and Nanog was observed in controls and in HP1γ knockdown cells (N1 and N2 cells). When considering markers of differentiation the situation was very different depending on the germ layers considered. The peaks and levels of expression of the neuroectodermal genes Sox1, Nestin, Musashi, were similar in the three populations. The same was true for the mesodermal markers Brachyury, Eomes And Mixl1. The situation was completely different for the endodermal markers Gata4, Gata6, Cxcr4, Pdgfrα presenting a tendency to lower expression levels at the time as the maximum was reached in control cells. These results thus indicate that the low level of HP1γ impairs the differentiation of embryoid bodies toward the endoderm. To further explore the role of HP1γ in the orientation of ESC differentiation we then used retinoic acid to induce a rapid differentiation, preferentially toward neuroectoderm and to a lesser extent to endoderm (Figure 7). The analysis by RT-qPCR showed that the expression of the pluripotency markers Oct4 and Nanog decreased rapidly and similarly in shHP1γ and control cells at day 2. The expression of the neuroectoderm-associated genes (Fgf5, Sox1 And Nestin) and of the mesoderm associated genes (Tbx2 and Flk1) followed similar kinetics in controls and HP1γ knockdown cells but their expression was enhanced in repressed cells. Both sets of sh RNA against HP1γ gave the same results in spite of uneven efficiency. In contrast, the expression of the endodermal markers Gata4, Gata6, Foxa2 and Hnf1 was not increased, showing similar or lower levels in sh HP1γ cells.

Altogether these results show that knockdown of HP1γ cells enhances the early commitment and drives differentiation in a way that limits the expression of endodermal markers in comparison with other lineages in both embryonic and retinoic acid differentiated cells.

### HP1γ is not regulated at the protein level during differentiation

**Figure 8:**
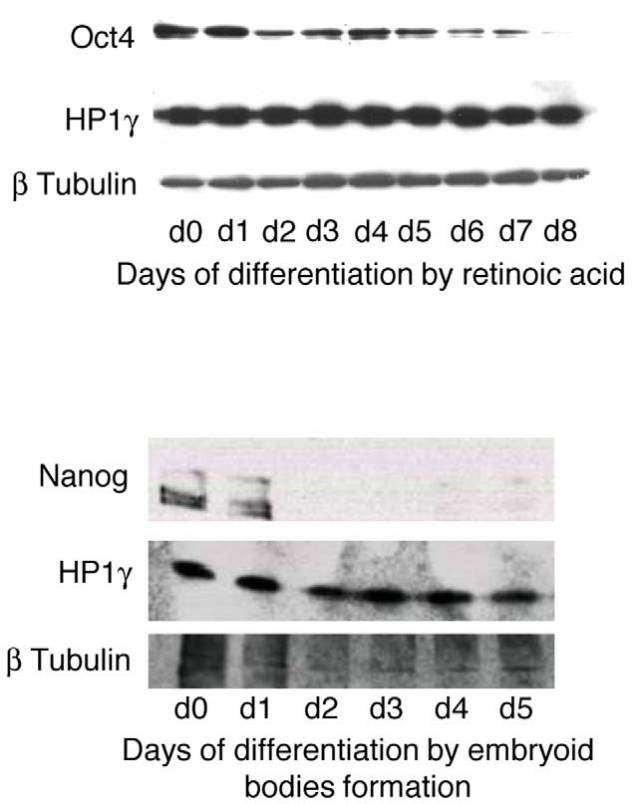
HP1γ protein level remains constant during ESC differentiation. ESC cells were induced to differentiate using 100 nM retinoic acid for 1 to 8 days (upper figure) or by embryoid bodies formation for 1 to 5 days (lower figure). The expression of the HP1γ, Oct4 and Nanog proteins were observed by western blot. Beta-tubulin was used as a protein loading control.

Since the expression level of HP1γ affects the ability of ESCs to differentiate, we wondered whether HP1γ might be regulated in a differentiation-dependent manner. The differentiation of ESCs was followed by a decrease in Oct4 protein levels when induced by retinoic acid, or by a decrease in Nanog protein levels when induced by embryoid bodies formation, as observed by western blot analysis. Within the same time periods, no change in the level of HP1γ protein was observed (Figure 8), indicating that the activity of HP1γ in ESCs was not controlled at this level.

## Discussion

Numerous reports have described the major epigenetic modifications associated with the differentiation of ESC, but very little is known about the epigenetic regulators that interpret these chromatin marks and control the switch from the pluripotent state to the differentiation programs. We have focused our interest on the chromatin associated protein HP1γ protein that, in addition to its role in the recruitment of transcriptional regulators, is associated with both the negative [37] and positive [29], [30], [32] regulation of gene transcription in the euchromatic regions [31] undergoing profound change during differentiation [8], [9].

Using an RNA interference approach to study the role of HP1γ in ESC, the results presented here show that HP1γ is involved in the self-renewal of ESC, in their commitment to differentiation and in the orientation of differentiation.

In proliferating conditions ESC repressed for HP1γ have a reduced proliferation rate. However they can be expanded, cultivated during this study with no limitation of time and do not show obvious morphological change. We rejected any cell cycle arrest or accumulation of dead cells as possible causes of the decreased proliferation rate. It has been reported that the knockdown of both HP1α and HP1γ by RNAi in HeLa cells abolishes the localization of the HP1-interacting kinetochore protein hMis12, resulting in aberrant chromosome segregation [38]. However, the authors have pointed out some functional redundancy between the two HP1 isoforms. In ESC, the presence of HP1α certainly prevented the cells from undergoing massive death due to mitotic catastrophe. We also failed to detect any increase of the β-galactosidase activity characteristic of senescent cells and expected if the protection of ESC from ageing was disrupted [39], [40] (data not shown). Therefore the self-renewal decrease of cells knocked-down for HP1γ results probably from a slowing-down of the cell cycle as a consequence of the deregulation of direct or indirect HP1γ target genes. Indeed, some cell growth regulators were misregulated upon HP1γ knock down. Hence, the function of HP1γ in ESC appears to be the control of self-renewal shown to result from the inhibition of differentiation inducing signaling [41]. It is noteworthy that this impact of HP1γ on proliferation seems to be restricted to highly self-renewing cells. Indeed, it has been shown that siRNA against HP1γ decreases the proliferation of cancer cell lines but not of noncancer-derived cell lines [42].

In ESC, as a probable consequence of the self-renewal defect, HP1γ depletion was found to disturb the balance between self-renewal and differentiation. First, the sporadic expression of differentiation genes that is inherent to ESC populations [11] was increased in HP1γ knocked-down cells, in correlation with an increased proportion of cells activating the promoter of the differentiation gene Brachyury. Second, an increased propensity to commit into differentiation was observed when the LIF that controls ESC maintenance was removed. However, in proliferating conditions the expression of several pluripotency markers, among which Oct4, Sox2, Nanog, Rex1 and SSEA1, was not affected, indicating that HP1γ depletion did not induce the spontaneous differentiation of ESC. The concomitant expression of pluripotency and differentiation makers along with proliferation defects show some similarities with the phenotype recently described for another chromatin associated protein, the histones acetyl transferase Tip60-p400 [13] that associates with Nanog.

Similarly shHP1γ cells share some common features with the reported phenotype of Nanog knockout ESC, which also remain self-renewing but have a reduced proliferation rate and an increased sensitivity to LIF privation [4], [5], [43]. This observation suggests that like Nanog, HP1γ may serve to block the transition toward differentiation. Whether the low proliferation rate of HP1γ knocked-down ESC is the cause or the consequence of the sporadic expression of differentiation genes is not elucidated here. It cannot be excluded that HP1γ contributes to the repression of these differentiation genes. It is however intriguing that among the restricted number of genes reproducibly misregulated by HP1γ knock down in ESC (less than 0.1%), a strong proportion is associated with the control of cell proliferation and not with differentiation, suggesting that the increased and sporadic expression of differentiation markers in ESC is the indirect consequence of the proliferation defect. It is of note that self-renewal is controlled by the classical Oct4/Sox2/Nanog set of transcription factors involved in differentiation blockage but also by another group of genes that directly regulate targets involved in cell cycle and cell survival [44]. This situation indicates that the ESC proliferation rate probably results from the cumulative effect of direct and indirect regulation pathways. The analysis of the transcripts in early-differentiating ESC indicated that the depletion of HP1γ mostly amplifies the variations of genes expression that are also targeted in control cells. In contrast, the genes specifically misregulated by HP1γ knock down during differentiation represent a minority. This expression pattern reinforces the idea that the function of HP1γ is to block the commitment of ESC toward differentiation.

Another important consequence of HP1γ depletion in ESC is to disadvantage differentiation toward the endoderm when compared with the neuroectoderm and/or the mesoderm pathways that in contrast were amplified. Further illustrating this bias, we observed that in proliferating conditions, markers of the three germ layers were detectable but only those of the neuroectoderm and the mesoderm were sporadically increased in cells with low level of HP1γ. These results indicate that in addition to its role in the balance between self-renewal and differentiation, HP1γ is required to manage the endoderm differentiation.

HP1 proteins are known to interact with numerous proteins, being described as a docking platform for some transcription factors [45]. In ESC HP1γ is abundantly expressed and its expression remains constant following differentiation induction, supporting the existence of non-transcriptional mechanisms to specify the different functions of HP1γ in ESC and upon differentiation such as post-translational modifications or the interaction with distinct binding proteins. In embryonic carcinoma cells the interaction between the transcription factor TIF1β and HP1 proteins sustains endoderm differentiation and is essential for late endoderm formation [46]. It can thus be assumed that the interaction between HP1γ and TIF1β accounts for the phenotype described here on the orientation of differentiation.

To conclude, our results support the growing evidence that a group of epigenetic regulators including HP1γ, Tip60/p400 [13] and Chd1 [12], are involved in the regulation of ESC properties by acting on ESC selfrenewal and controlling the expression of differentiation genes. The phenotype associated with HP1γ depletion is an intermediate between those described for the two other proteins by two aspects. First, the supression of HP1γ slows down the cell cycle without decreasing the expression of pluripotency genes in ESC as described for Tip60/p400 [13]; second, the depletion of HP1γ orientates the differentiation as described for Chd1 [12]. Both Tip60/p400 and Chd1 recognize methylated H3K4 that are associated with active genes [47] and are abundantly represented in ESC chromatin [20] [21].

Differently from the two other chromatin binding proteins, HP1γ recognizes histone marks associated with heterochromatin formation and gene silencing. The results presented here suggest that pathways regulating the function of apparently antagonistic epigenetic regulators converge to maintain ESC identity, notably in their ability to give rise to a well-balanced pattern of differentiation between the three germ lineages. Understanding how HP1γ is regulated to sustain specific functions will provide insight into the underlying network.

## Materials and Methods

### Plasmids and production of lentiviral vectors

The short hairpin (sh) lentiviral vectors used were Mission-shRNA (derived from pLKO.1-puro) purchased from Sigma (MISSION® shRNA; Sigma-Aldrich, St. Louis). Self-inactivating HIV-1-derived vectors were generated by the transient transfection of 293T cells as previously described [48] using pCMV-G [49] and pCMVdeR8.91 [50]. Viral supernatants were harvested 24 hours after transfection and then filtered on 0.45 µm porosity filter.

### ES cell culture, retroviral infection and differentiation

CGR8 mouse ESC were grown on gelatin coated dishes in proliferation medium (Glasgow's modified Eagle's medium supplemented with 10% fetal calf serum (FCV) (PerbioScience, Berbieres, France), 10% of non essential amino acids (Invitrogen Carlsbad, CA), 10% of sodium pyruvate (Invitrogen) and 1000 U/ml LIF (Abcys, Paris, France).

For lentiviral infection, CGR8 cells were plated at a density of 2.10^5^ cells per 4 cm dish and cultivated for 24 hours. The viral supernatant was then added and ESC were cultivated for 24 hours before selection by puromycin (1 µg/ml) (Invitrogen) for 4 days. Resistant colonies were dissociated and pooled for amplification in the presence of puromycin for 2 days before all analysis.

For retinoic acid (RA)-induced differentiation, 5.10^5^ cells were seeded per 4 cm dish. RA (Sigma Aldrich) was added at a final concentration of 10^−7^ M 24 hours after plating (referred to as day 0) in ES cells differentiation medium that is the same as proliferation medium but without LIF and with 5% FCV.

For EB-induced differentiation, proliferating cells were seeded in differentiation medium (final density 6.10^5^ cells in 10 ml) and allowed to float in non-adherent Petri dishes during the indicated times.

### Decreasing LIF concentration assay

Cells in exponential phase of growth were seeded at very low density (10^4^ cells in a 3.5 cm diameter dish) and cultured for 5 days before alkaline phosphatase staining and analysis.

### Detection of alkaline phosphatase activity

Cells were fixed for 30 min at 4°C (1.5% formaldehyde and 0.5% glutaraldehyde in PBS). After washes with PBS, the cells were stained using a diluted solution of NBT/BCIP (Roche) prepared according to the manufacturers' instructions. Colonies were observed and counted using an inverted microscope (Axovert 135, Zeiss).

### Measurment of cell proliferation by BrdU incorporation assay

ES cells were seeded in 96-well dishes at a density of 2×10^3^ cells/well and cultivated overnight to obtain 40% confluence. BrdU from the ELISA BrdU kit (Roche Applied Science, Basel, Switzerland) was added to the wells for the last 8 hours of culture. BrdU incorporation was measured by colorimetry according to the manufacturer's instructions.

### Cell cycle analysis

Exponentially growing ESC were refed with fresh medium and incubated for 2 hours. BrdU was then added at a final concentration of 50 µM and the cells were incubated for 40 minutes. The cells were trypsinised and 5 million of them were fixed and labeled with propidium iodide and anti-BrdU antibody as described [51]. Flow cytometry analyses were performed using FACS (FACScalibur 4C + HTS; BD Biosciences). Data acquisition was performed using the CellQuest Pro software (BD Bioscience).

### Cell death detection

The cells were cultivated overnight. The supernatant was removed and the cells were dissociated using trypsine/EDTA (Invitrogen). After centrifugation and washing with PBS, Annexin V labeling (Bender Med Systems) was performed according to the manufacturer's instructions. Just before analysis by flow cytometry BrdU was added at a final concentration of 1 µg.mL^−1^.

### Detection of Stem Cell-Specific Embryonic Antigen-1 expression

Dissociated cells were labeled using PE-labeled anti-SSEA1 antibody (anti-SSEA1-phycoerythrin, R&D Systems Inc., Minneapolis) applied for 45 min on ice. After washing, the fluorescence intensity was determined by flow cytometry.

### Measurment of the Brachyury promoter activity

The cells were infected with a lentivirus containing a GFP transgene under the control of the Brachyury gene promoter (Brachyury-eGFP, Addgene). After one week of culture, the cell line was transduced with short hairpin vectors directed against HP1γ (N1 and N2) or control. Transduced cells were selected by culture in the presence of puromycin for a week before analysis by flow cytometry.

### Real Time Quantitative Polymerase Chain Reaction (RT -QPCR)

RNA was extracted using an RNeasy kit with on-column DNase digestion, according to the manufacturer's recommendations (Qiagen, Hilden, Germany). Reverse transcription was carried out with 1 µg of RNA and SuperScript II (Invitrogen) according to the manufacturers' recommendations.

Real-Time PCR was performed using the MXP-300P PCR-system (Stratagene, Amsterdam, Netherlands) and Mix-Quantitect SYBR Green (Qiagen, Hilden, Germany) as reagent. Regimens of 40 cycles at 95°C for 30 seconds, at 55°C for 1 minute and 72°C for 30 seconds were applied. Samples were run in duplicate and gene expression levels were calculated using Delta Delta Ct (http://www.gene-quantification.info/) normalized with the mouse 40S ribosomal protein S17 as housekeeping gene. The number of independent experiments performed is indicated in each figure legend.

### Oligonucleotide sequences

The oligonucleotide primers for PCR, listed in Table 2, were designed using the Primer3 software (http://frodo.wi.mit.edu/cgi-bin/primer3/primer3_results.cgi) and were purchased from MWG (Eurofins MWG Operon, Ebersberg, Germany). Qiagen Quantitec primers (Qiagen) were used for the RT-qPCR validation of cell growth-associated gene (Table S1).

**Table 2:** Oligonucleotide primers used in QPCR experiments.

Gene	Sense	Antisense
Brachyury	CCGGTGCTGAAGGTAAATGT	CCTCCATTGAGCTTGTTGGT
Cbx1	GTCAAGGGCAAGGTGGAATA	CCTCGTGGCTTTTCTGACTC
Cbx3	GAGATGCTGCTGACAAACCA	GCTCCTCGTAGAAGGCAATG
Cbx5	TCTGTCATTGCCACTTGAGC	CCCTTCCTTCACCACTGTGT
Cxcr4	TCCTGCCCACCATCTACTTG	CTTTTCAGCCAGCAGTTTCC
Eomes	GGCAAAGCGGACAATAACAT	AGCCTCGGTTGGTATTTGTG
Fgf5	CGCTTTGACTGGAACTAAAC	GAATGCTAACCATCCTCAAA
Flk1	GTAAAAGCAGGGAGTCTGTG	GTGGTGGAAAGAACAACACT
Foxa2	TGGTCACTGGGGACAAGGGAA	CTGCAACAACAGCAATAGAGAACAAC
Gata4	CTGTGCCAACTGCCAGACTA	GCATCTCTTCACTGCTGCTG
Gata6	ACAGCCCACTTCTGTGTTC	TGGGTTGGTCACGTGGTACA
Hnf1	GATGTCAGGAGTGCGCTACA	CTGAGATTGCTGGGGATTGT
Mixl1	GCACGTCGTTCAGCTCGGAG	GTCATGCTGGGATCCGGAACGTG
Musashi1	CGGGGAACTGGTAGGTGTAA	ATGCTGGGTATTGGGATGCT
Nanog	AAGTACCTCAGCCTCCAGCA	GTGCTGAGCCCTTCTGAATC
Nestin	GAAGACCAGCAGGCGTTTAG	TCCTCTGCGTCTTCAAACCT
Oct4	CACGAGTGGAAAGCAACTCA	AGATGGTGGTCTGGCTGAAC
Pdgfr α	CAAGAGAGTGACTGGCCACA	CGGTTCCAGTACCTTCCAAA
Rex1	CGTGTAACATACACCATCCG	GAAATCCTCTTCCAGAATGG
Rs17	ATGACTTCCACACCAACAAGC	GCCAACTGTAGGCTGAGTGAC
Sox1	CACAACTCGGAGATCAGCAA	GTCCTTCTTGAGCAGCGTCT
Tbx2	CGAGGAGTCAGTCTATCCAG	ACCTCTACCCTATGCACCTT

### Western blots

Cytoplasmic proteins separated on 10% SDS-polyacrylamide gels were transfered on Hybond ECL membranes (GE Healthcare). Blots were submitted to Western analysis using the following antibodies: anti-HP1γ (clone 2MOD-1G6AS, Euromedex), anti-Oct-3/4 (clone H-134, Santa Cruz Biotechnology), anti-Nanog (ab-21603, Abcam), anti-β tubulin (clone TUB2.1, Sigma).

### Affymetrix GeneChip Assays

#### Experimental design

Three completely independent experiments were carried out, with three independent infections, cell cultures and EB experiments.

#### Processing of RNA

Biotinylated antisense cRNA for microarray hybridization was prepared using the GeneChip® One-Cycle target labeling kit and procedures from Affymetrix (Santa Clara, CA, USA). cRNA quantification was performed with a Nanodrop and quality checked with an Agilent 2100 Bioanalyzer (Agilent technologies, Inc, Palto Alto, CA, USA).

#### Array hybridization and scanning

Microarray analyses were performed using high-density oligonucleotide arrays (Mouse Genome 430 2.0 Array, Affymetrix). Biotinylated cRNA (15 µg) was fragmented and hybridization on the chip was performed following the Affymetrix protocol (http://www.affymetrix.com). Washing and staining were performed in a Fluidics Station 450 (Affymetrix). The arrays were scanned with a confocal laser (Genechip Scanner 3000, Affymetrix) and analysed with Expression Console Software (Affymetrix).

#### Microarray data analysis

The results were filtered using Genespring 7.3.1 (Agilent). A first selection of genes was performed by pairwise comparisons between shCTR and shN2 (table 1) or between shCTR, shN2, shCTR-EB36h and shN2-EB36h (Figure 5). Each sample from one group was compared with each sample from the other group, and only genes showing a fold change ≥1.8 between groups were retained. A gene was considered differentially expressed only if it met the above criteria in all pairwise comparisons and if the detected signal was above the background in at least one of the compared groups, thereby carrying a statistically significant absolute call of ‘present’ or ‘marginal’ in all samples.

A list of genes associated with differentiation was generated with MedscanReader 2.2 (Ariadne Genomics) and Pathway Studio 6.2 (Ariadne Genomics) software that enables the automated extraction of information from scientific text (list displayed as supplementary data). Genes that were differentially expressed or common to the generated list were clustered into a tree based on Pearson correlation with the average linkage used as a clustering algorithm.

#### Microarray validation by RT-qPCR

To validate the results of the microarray analysis, seven genes of biological significance were subjected to RT-qPCR and their expression levels were measured (Table S2).

All microarray data is MIAME compliant and the raw data has been deposited in Array Express at http://www.ebi.ac.uk/microarray-as/ae/. Accession number is E-MEXP-2238.

## Supporting Information

Table S1 Validation of microarray fold change by RT-qPCR for genes associated with cell growth. (RTF)

Table S2 Microarrays validation by RT-qPCR. (RTF)

File S1 List of the genes displayed in Figure 5B. (RTF)
