# Supplementary material for: Role of the Epigenetic Regulator HP1γ in the Control of Embryonic Stem Cell Properties
Source: PLoS One. 2010 Nov 15;5(11):e15507. doi: 10.1371/journal.pone.0015507 (PMC2981578; doi:10.1371/journal.pone.0015507)
Supplement: Table S1 — Validation of microarray fold change by RT-qPCR for genes associated with cell growth. (RTF) [file pone.0015507.s001.rtf]

Table S1. Validation of microarray fold change by RT-qPCR for genes associated with cell growth.
	Array ratio 	Array Standard Error 	Qpcr ratio 	Qpcr Standard Error 	
Nrp2	0.46	0.06	0.32	0.08	
Aldh3a1	0.26	0.07	0.22	0.02	
Calml4	0.36	0.03	0.28	0.01	
Ckmt1	0.40	0.04	0.35	0.03	
Gpx2	0.27	0.09	0.18	0.02	
Pla2a	0.29	0.04	0.18	0.02	
Performed on Sh39 J0 samples
