# Supplementary material for: Role of the Epigenetic Regulator HP1γ in the Control of Embryonic Stem Cell Properties
Source: PLoS One. 2010 Nov 15;5(11):e15507. doi: 10.1371/journal.pone.0015507 (PMC2981578; doi:10.1371/journal.pone.0015507)
Supplement: Table S2 — Microarrays validation by RT-qPCR. (RTF) [file pone.0015507.s002.rtf]

Table S2. Microarrays validation by RT-qPCR

	Sh39 J0	ShCTR EB36h	Sh39 EB36h	
	Array ratio	Array Standard Error	qpcr ratio	Qpcr Standard Error	Array ratio	Array Standard Error	qpcr ratio	Qpcr Standard Error	Array ratio	Array Standard Error	qpcr ratio	Qpcr Standard Error	
HP1γ	0.20	0.10	0.09	0.02	1.03	0.06	1.21	0.16	0.37	0.04	0.27	0.05	
HP1α	1.26	0.35	0.96	0.21	1.52	0.35	1.75	0.42	1.54	0.25	1.61	0.56	
HP1β	1.07	0.93	1.02	0.33	3.36	0.35	2.06	0.54	2.89	1.80	1.82	0.62	
Oct4	1.01	0.08	0.86	0.09	0.89	0.08	1.20	0.06	0.86	0.09	0.85	0.18	
Nanog	1.12	0.07	1.02	0.07	0.50	0.12	0.57	0.06	0.30	0.08	0.23	0.01	
Sox2	0.93	0.05	0.91	0.12	0.88	0.03	1.16	0.06	0.78	0.07	0.82	0.15	
Rex1	0.96	0.11	1.03	0.01	0.67	0.06	0.72	0.07	0.34	0.04	0.24	0.03	
