# Supplementary material for: Role of the Epigenetic Regulator HP1γ in the Control of Embryonic Stem Cell Properties
Source: PLoS One. 2010 Nov 15;5(11):e15507. doi: 10.1371/journal.pone.0015507 (PMC2981578; doi:10.1371/journal.pone.0015507)
Supplement: File S1 — List of the genes displayed in Figure 5B. (RTF) [file pone.0015507.s003.rtf]

Text S1: List of the genes displayed in Figure 5B.

Accession number	Gene Name	
BB333334	W; Bs; Fdc; Ssm; CD117; c-KIT; Tr-kit	
M75135	Glut3; C78366; Glut-3; AA408729; AL023014; AL024341; AU040424	
X69698	Glut3; C78366; Glut-3; AA408729; AL023014; AL024341; AU040424	
BM115022	SCG10; Stmb2; Scgn10; AI159727	
BM115022	SCG10; Stmb2; Scgn10; AI159727	
BI658627	sFRP-1; AW011917; AW107218; AW742929; 2210415K03Rik	
NM_008598	AGT; Agat; AI267024; MGC107020	
AV239350	CPS; D1Ucla3; 4732433M03Rik	
NM_021346	TZF; 2610034E08Rik; D530032D06Rik; Zfp318	
BB753447	AP-2rep; AI225908; AI315654; 2700063E05Rik; B130052C06Rik	
AV214969	Ebaf; Lefta; AV214969; MGC98569; 6030463A22Rik	
BB045423	D5Ertd189e; Tbx3	
BE688087	GABAbR1; bM573K1.1	
NM_008416	Junb	
NM_009556	Rex1; Rex-1; Zfp-42; MGC107378	
NM_009506	VEGF-C; AW228853	
AW228853	VEGF-C; AW228853	
BC012697	Slc25a8	
AK010384	LAP; LAPEP; AA410100; 2410015L10Rik	
AW108044	Slc25a8	
AI987929	RTP; DRG1; NMSL; Ndr1; TDD5; CAP43; CMT4D; HMSNL; PROXY1	
AV309418	RTP; DRG1; NMSL; Ndr1; TDD5; CAP43; CMT4D; HMSNL; PROXY1	
AI987929	RTP; DRG1; NMSL; Ndr1; TDD5; CAP43; CMT4D; HMSNL; PROXY1	
AF176530	Fbx15; ecat3; AU019763	
BB089170	VEGF-C; AW228853	
NM_008452	Lklf	
BC004617	AW495861; 5730403J10Rik	
NM_007657	Tspan29	
BC008626	CD54; Ly-47; Icam-1; MALA-2; MGC6195	
BB206220	Kid1; BC026639; MGC27707; MGC37517	
BE853286	3110023F10Rik	
AK010332	ENK; ecat4; 2410002E02Rik	
AA543734	D5Ertd189e; Tbx3	
BI659446	Csnk1a; MGC29354; MGC30571; 2610208K14Rik; 4632404G05Rik; 5430427P18Rik	
BM114422	GABAbR1; bM573K1.1	
AI325183	Aprf; AW109958; 1110034C02Rik; Stat3	
AK013312	CycB2	
AK004083	Aprf; AW109958; 1110034C02Rik; Stat3	
NM_013496	Rbp-5; CrabpI; Crabp-1; AI326249	
BI465857	CKLF; IKLF; Bteb2; 4930520J07Rik	
NM_009760	Nip3	
BB398886	PAR4; Par-4	
BG069413	EZF; Zie; Gklf	
BG069413	EZF; Zie; Gklf	
BF467211	AI747189; AU018915	
U50279	VPF; Vegf; VEGF-A; VEGF120; Vegfa	
AK012780	MKBP; 27kDa; HSP27; 2810021G24Rik	
AK010420	Myd118; AI323528	
AI323528	Myd118; AI323528	
NM_009505	VPF; Vegf; VEGF-A; VEGF120; Vegfa	
NM_022721	Fz5; 5330434N09Rik	
NM_011498	CR8; Dec1; Clast5; Sharp2; Stra13; Stra14; C130042M06Rik	
BB414515	Glut3; C78366; Glut-3; AA408729; AL023014; AL024341; AU040424	
L16846	AW546738	
NM_009263	OP; Bsp; Eta; Opn; Ric; BNSP; BSPI; Opnl; Apl-1; ETA-1; Spp-1; AA960535; AI790405; minopontin	
NM_011546	BZP; ZEB; MEB1; Nil2; Tcf8; AREB6; Tcf18; Zfhep; Zfx1a; Zfx1ha; [delta]EF1; 3110032K11Rik	
NM_009035	CBF1; RBP-J; RBPjk; Igkjrb; Igkrsbp; AI843960	
NM_009035	CBF1; RBP-J; RBPjk; Igkjrb; Igkrsbp; AI843960	
AK010379	Lrf; FBI-1; Zbtb7; Pokemon; AI452336; 9030619K07Rik; 9130006G12Rik	
NM_008989	CAGER-1; ssCRE-BP; Pur-alpha	
NM_008604	NEP; CD10; CALLA; C85356; 6030454K05Rik	
AV174022	NEP; CD10; CALLA; C85356; 6030454K05Rik	
AI426862	CAGER-1; ssCRE-BP; Pur-alpha	
NM_010176	Fah	
BB763517	bw; mi; wh; vit; vitiligo; MGC124309; MGC124310	
BC019986	Cdx; Cdx-1	
U36502	STAT5; AA959963	
NM_007557	OP1	
BB728182	D5Ertd189e; Tbx3	
NM_010875	CD56; Ncam; E-NCAM; NCAM-1; NCAM-120; NCAM-140; NCAM-180	
AV343511	MP1; Ntup1; AA410010; mKIAA1104; 2310012C15Rik	
AB026497	MyoPDZ; MysPDZ	
BC018375	Hry	
BC017621	Kid1; BC026639; MGC27707; MGC37517	
NM_008197	H1fv; H1(0); MGC19309; MGC98218; MGC117919; D130017D06Rik	
BC003830	H1fv; H1(0); MGC19309; MGC98218; MGC117919; D130017D06Rik	
BB698413	CD56; Ncam; E-NCAM; NCAM-1; NCAM-120; NCAM-140; NCAM-180	
X65997	W; Bs; Fdc; Ssm; CD117; c-KIT; Tr-kit	
NM_013864	Ndr2; SYLD; AI182517; AU040374	
AV360881	TBX1; C85531; N4WBP3; 3222402J11Rik	
AI463328	DBK; Pkn; PAK1; PRK1; Stk3; Prkcl1; F730027O18Rik	
BB331017	Sox-1	
AV173869	CAST; 6430531D06; CAST1/ERC2	
NM_007408	ADPH; Adrp; AA407157	
BC014727	TAZ; C78399; 2310058J06Rik; 2610021I22Rik	
BC014727	TAZ; C78399; 2310058J06Rik; 2610021I22Rik	
AF326547	EP10; GAD67; Gad-1	
NM_008480	Lama; AA408497	
BB253137	Inhbb	
AV259665	MISIIR; Misrii	
AV209206	MISIIR; Misrii	
U36502	STAT5; AA959963	
AF426024	EI; EIA; LEI; PI2; MNEI; M/NEI; ELANH2; AI325983; MGC129309; 1190005M04Rik	
BC026422	Tgase1; 2310004J08Rik	
NM_022879	MLC2a; MYL2A; RLC-A; Mylc2a; MLC-2alpha	
NM_133926	AI505105; D6Ertd263e	
BB667216	VWD; F8VWF; AI551257; C630030D09; 6820430P06Rik; B130011O06Rik	
NM_009292	Stra8	
BM239162		
NM_019390	Lmna	
NM_013820	HKII; AI642394	
NM_007554	Bmp2b; Bmp2b1; Bmp2b-1	
NM_011446	Sox7	
D63423	Anx5; R74653	
AI266795	S100; S100a; AI266795	
X14480	Nid; entactin; nidogen-1; A630025O17; entactin-1	
X14480	Nid; entactin; nidogen-1; A630025O17; entactin-1	
BC008107	Clgi; Timp; TIMP-1; MGC7143	
AK008784	BB116197; 2010310L10Rik; 2210401D16Rik	
AW108044	Slc25a8	
NM_021882	gp87; gp100; Pmel17; D12S53Eh; D10H12S53E	
NM_007430	AHX; Ahc; Ahch; Dax1; DAX-1	
BC004695	Zfp64	
BC021637	gp110; Scard1	
NM_007639	Cd1a; Cd1d; CD1.1; Ly-38; AI747460	
BM224327	CD32; Fcgr2; Fcr-2; Fcr-3; Ly-17; LyM-1; Lym-1; FcgRII; Fcgr2a; Ly-m20; AI528646; Fc[g]RII; F630109E10Rik	
NM_008116	GGT; Ggtp; CD224; AI746379	
C80272	Ftf; LRH-1; D1Ertd308e	
NM_019394	MIA; Cdrap; CD-RAP	
W29605	Wnt-7b	
NM_010449	ERA1; Hox-1.6	
BG094302	AU024132; AU043124; Galpha13	
NM_009627	AM	
AV147875	MGC102095	
AV147875	MGC102095	
BC010197	CPH; fat; Cph1; Cph-1; R74677; MGC7101	
AV026617	c-fos; D12Rfj1	
BC018354	M32; HP1g; MGC118084; LOC382769; LOC544944	
NM_008508	S77319; AI036317	
BB311687	Xt; Bph; Pdn; add; AI854843; AU023367	
AV240088	go; Fgf-5; angora	
AB016516	go; Fgf-5; angora	
AI323543	Mat1; PEA-15; Pkcs15; Pea15	
BC017609	E130306E05Rik	
NM_013916	Ha1r; RLIM; AL022832; AW743871	
BB706377	Tex17; Lin-28; Lin28a; AL024421	
M20480	Nfl; NF-L; NF68; CMT2E; AI847934	
U36203	sno; Skir; SnoN; SnoN2; sno-dE3; 9130011J04Rik	
AA214868	TEP1; MMAC1; AI463227; 2310035O07Rik; A130070J02Rik	
U43884	Idb1; AI323524; D2Wsu140e	
U30244	Epl5; ELF-2; Eplg5; Htk-L; Lerk5; LERK-5; NLERK-1	
AW493905	5730485F04Rik	
BC001991	Se-P; selp; AU018766; D15Ucla1	
BB751459	AI467640; AW121680; mKIAA1077	
AK004119	SemE; Semae; 1110036B02Rik	
BF019883	Idb2; C78922; AI255428	
BF456404	Paka; PAK-1; AW045634	
M55512	Wt-1; D630046I19Rik	
BG065255	Otf6; Scip; Tst1; Oct-6; Test1; Tst-1	
NM_009876	CDKI; Kip2; p57Kip2; AL024410; p57(kip2)	
BB795491	W91664; AU015806; Dnmt3a	
AK017686	Hox-2.9; MGC124123	
NM_018826	Irx5	
NM_010068	MGC124407; Dnmt3b	
NM_011141	Otf6; Scip; Tst1; Oct-6; Test1; Tst-1	
BQ174638	Ank-2; AI835472; AW491075; Ankyrin-2; Ankyrin-B; ankyrin B; ankyrin>B<; Ank2	
U30244	Epl5; ELF-2; Eplg5; Htk-L; Lerk5; LERK-5; NLERK-1	
AV226212	3930401C23	
M64279	Bmi1; Bmi-1; AW546694	
BC022107	CDHN; Ncad; N-cadherin	
M64279	Bmi1; Bmi-1; AW546694	
NM_010014	scm; scr; yot; scrambler; C630028C02Rik; Dab1	
L27453	Pbx-1; D230003C07Rik; Pbx1	
M63801	Cx43; Npm1; Cnx43; Gja-1; AU042049; AW546267; Cx43alpha1; connexin43	
BB795491	W91664; AU015806; Dnmt3a	
BB795491	W91664; AU015806; Dnmt3a	
BE989096	Ah; In; Ahh; Ahre	
BF456404	Paka; PAK-1; AW045634	
BB037416	spdh; Hox-4.8	
AV232123	Tfm; AW320017	
BB371406	Fzd10; Mfz10; Mfz10a; AW456835	
BG970109	C77966; C80098; C81607; Lamb-1; D130003D08Rik	
BB202790	MGC124407; Dnmt3b	
BC003851	CAP1; mCAP1; C79772; AI313909; 2410039E18Rik	
NM_009290	Wnt8d; Stra11; Wnt-8A; Wnt-8D	
BC021623	Ap2; AP-2; Ap2tf	
BB371406	Fzd10; Mfz10; Mfz10a; AW456835	
BB297502	5730485F04Rik	
BC005718	AL024037	
BB065799	AI467640; AW121680; mKIAA1077	
BG067039	CAG-2; C81439; D0Kist1; mKIAA1071; E230009N18Rik	
BB417145	5730485F04Rik	
U30244	Epl5; ELF-2; Eplg5; Htk-L; Lerk5; LERK-5; NLERK-1	
BC002064	OSF; HARP; HBBN; HBNF; Osf1; Osf-1; HB-GAM; HBGF-8	
BG966339	Lec1; Gm619; Lphh1; AI450192; MGC38872; mKIAA0786	
BC003264	ATX; Npps2; Pdnp2; Autotaxin; PD-Ialpha	
BC006640	PBSF; Sdf1; TLSF; SDF-1; Sdf1a; Sdf1b; TPAR1; Scyb12; TLSF-a; TLSF-b; AI174028; PBSF/SDF-1; Cxcl12	
BG966339	Lec1; Gm619; Lphh1; AI450192; MGC38872; mKIAA0786	
NM_010329	T1a; Gp38; OTS-8; PA2.26; T1alpha	
NM_008173	GR; Grl1; Grl-1	
NM_007394	ALK2; Acvr; Alk8; SKR1; Alk-2; Tsk7L; ActR-I; ActRIA; Acvrlk2; D330013D15Rik	
NM_010217	Hcs24; Fisp12; fisp-12	
BB202655	NrCAM; mKIAA0343; C030017F07Rik	
AK013416	Ttg2; Rbtn2; Rbtn-2; Rhom-2	
BG873440	Bek; Fgfr7; Fgfr-2; Fgfr-7; KGFRTr; AU043015; AW556123; MGC102519; Fgfr2	
BG970109	C77966; C80098; C81607; Lamb-1; D130003D08Rik	
AV359819	Htu; Ser-1; slalom; Serrate-1; Headturner	
BE825056	end1; AI836553; 1110038H03Rik; 6230403H02Rik	
BM508495	end1; AI836553; 1110038H03Rik; 6230403H02Rik	
BM508495	end1; AI836553; 1110038H03Rik; 6230403H02Rik	
AV330726	Cx43; Npm1; Cnx43; Gja-1; AU042049; AW546267; Cx43alpha1; connexin43	
BB142324	Cx43; Npm1; Cnx43; Gja-1; AU042049; AW546267; Cx43alpha1; connexin43	
M63801	Cx43; Npm1; Cnx43; Gja-1; AU042049; AW546267; Cx43alpha1; connexin43	
BB039269	Cx43; Npm1; Cnx43; Gja-1; AU042049; AW546267; Cx43alpha1; connexin43	
BC022107	CDHN; Ncad; N-cadherin	
BC003264	ATX; Npps2; Pdnp2; Autotaxin; PD-Ialpha	
U80888	CAG-2; C81439; D0Kist1; mKIAA1071; E230009N18Rik	
AW546010	Xt; Bph; Pdn; add; AI854843; AU023367	
BB371406	Fzd10; Mfz10; Mfz10a; AW456835	
AI596632	AW046354; AW549872	
BB080177		
BB080177		
NM_016889	IA-1	
AU021035	Hspg1; Synd2; AA960457; syndecan-2	
AV238324	LPb3; S1P3; AI132464	
AI385532	TSP1; TSP-1; tbsp1; Thbs-1	
BC005453	Nmyc; N-myc; Nmyc1; Nmyc-1; c-nmyc	
BC006622	AF311610; AI385680; AI661372; K-glypican; 9530073D23Rik	
AK005032	Mir; 1300017E09Rik	
BB698679	Alk6; ALK-6; Acvrlk6; BMPR-IB; CFK-43a; AI385617; AV355320	
NM_133654	AU040960	
NM_008003	Fgf15	
X70514	Tg.413d	
X70514	Tg.413d	
NM_010496	Idb2; C78922; AI255428	
BG072739	end1; AI836553; 1110038H03Rik; 6230403H02Rik	
NM_019389	NG2; hdf; PG-M(V0); PG-M(V1); 9430051N09; 5430420N07Rik	
BC021876	JAM; Jcam; JAM-1; JAM-A; Jcam1; Ly106; ESTM33; AA638916; 9130004G24	
BB815530	Gb; SF; Sl; Clo; Con; Mgf; SCF; SLF; Steel; contrasted	
NM_010068	MGC124407; Dnmt3b	
AF290209	PC; Ly102; Pclp1; AW121214	
BB815530	Gb; SF; Sl; Clo; Con; Mgf; SCF; SLF; Steel; contrasted	
BB409331	Jacop; AI503810; 4933421H10Rik; 9930020M10Rik	
BC018383	NF-66; AV028420; MGC25352	
BC018383	NF-66; AV028420; MGC25352	
BC018383	NF-66; AV028420; MGC25352	
NM_011254	Crbp; CRBPI; Rbp-1	
BQ176915	Isl1	
BB425719	ASH1; Mash1; AI225900	
NM_007439	Tcrz; CD246	
NM_008783	Pbx-1; D230003C07Rik; Pbx1	
BB750646	HD9; HD7B; HDRP; Mitr; Hdac7b; AV022454; mKIAA0744; D030072B18Rik	
Y19235	p73	
BC016426	msh; Hox7; Hox-7; Hox7.1; AA675338; AI324650	
BB148302	Tfm; AW320017	
NM_009144	Sdf5; AI851596	
NM_010437	MIBP1; Shn-2; Schnurri-2	
NM_008716	AW229011	
BB361162	ZIC; ZNF201	
NM_008259	Hnf3a; Tcf3a; Hnf-3a; Tcf-3a	
AV175389	IGFBP-3; IGgfbp3; AI649005	
NM_008216	Has2	
BB468410	IA-1	
NM_008935	Prom; AC133; CD133; Proml1; 4932416E19Rik	
NM_008216	Has2	
NM_013655	PBSF; Sdf1; TLSF; SDF-1; Sdf1a; Sdf1b; TPAR1; Scyb12; TLSF-a; TLSF-b; AI174028; PBSF/SDF-1; Cxcl12	
NM_023580	Eph; Esk; AL033318; 5730453L17Rik	
BB444134	Fst	
NM_010052	FA1; ZOG; pG2; Peg9; SCP1; Ly107; pref-1; AW742678	
NM_130449	SRCL; CL-P1; Scara4	
D78264	AMY; Noe1; OlfA; AW742568; Pancortin; Pancortin3	
NM_008393	AI894186	
BC018397	Crabp-2; CrabpII; AI893628	
AI649005	IGFBP-3; IGgfbp3; AI649005	
NM_010056	AI385752; Dlx5	
NM_021543	Papc; 1700080P15Rik	
NM_009801	CAII; Car-2; Ltw-5; Lvtw-5; AI131712	
BB074430	Peg6; AI528698	
AW743020	Peg6; AI528698	
AV124445	Peg6; AI528698	
NM_009866	Cad11; MGC25339; 2810038F24; 2610005L07Rik	
AU021035	Hspg1; Synd2; AA960457; syndecan-2	
BB076893	Papc; 1700080P15Rik	
AV315205	BEN; SC1; MuSC; CD166; AI853494; DM-GRASP; MGC27910	
BB096079	GR; Grl1; Grl-1	
BB549310	AMY; Noe1; OlfA; AW742568; Pancortin; Pancortin3	
BB453775	JNK3; Serk2; JNK3B1; JNK3B2; p493F12; p54bSAPK; SAPK(beta)	
AK014844	Mvh; VASA; AV206478	
AF215668	GOS8	
NM_007556	Vgr1; Vgr-1; D13Wsu115e	
NM_010054	Dlx-2; Tes-1; AW121999	
NM_013601	Hox8; Hox-8; Hox8.1; BB122635	
X58380	pg; Hmgic; pygmy; HMGI-C; MGC103393; 9430083A20Rik; Hmga2	
NM_008957	Ptc; mes; Ptc1; Ptch; A230106A15Rik	
BM251152	NG2; hdf; PG-M(V0); PG-M(V1); 9430051N09; 5430420N07Rik	
BE825056	end1; AI836553; 1110038H03Rik; 6230403H02Rik	
BC013446	PPR; Pthr; PTH1R	
M27130	Ly-24; Pgp-1; HERMES; AW121933; AW146109	
BB177836	p35; Cdk5r; D11Bwg0379e	
X96585	C130088N23Rik	
BB234940	Cak; Nep; PTK3A; CD167a; AI323681; 6030432F18	
AI874681	Cak; Nep; PTK3A; CD167a; AI323681; 6030432F18	
BG083485	Sox-4; AA682046	
AI428101	Sox-4; AA682046	
AI428101	Sox-4; AA682046	
BB345784	IRS-1	
AI428101	Sox-4; AA682046	
AV156352	Lef-1; AI451430; 3000002B05	
BB480970	Pbx-1; D230003C07Rik; Pbx1	
AV316207	MGC124407; Dnmt3b	
BB441213	Ha1r; RLIM; AL022832; AW743871	
BG074676	Ha1r; RLIM; AL022832; AW743871	
BB040049	Ptc; mes; Ptc1; Ptch; A230106A15Rik	
AV071536		
BB656631	end1; AI836553; 1110038H03Rik; 6230403H02Rik	
BB034265	GOS8	
AY057913	Bdnf	
BG073383	Hox-2.7	
AW555326	bnb; Smoh; D13Mgi8; E130215L21Rik	
NM_013689	Tec	
BC006728	Myc2; Nird; Niard; c-myc; AU016757	
NM_009829	Vin1; Vin-1; C86853; AI256817; BF642806; 2600016F06Rik	
AV310588	Vin1; Vin-1; C86853; AI256817; BF642806; 2600016F06Rik	
AK007904	Vin1; Vin-1; C86853; AI256817; BF642806; 2600016F06Rik	
BQ175880	Vin1; Vin-1; C86853; AI256817; BF642806; 2600016F06Rik	
NM_009829	Vin1; Vin-1; C86853; AI256817; BF642806; 2600016F06Rik	
NM_009829	Vin1; Vin-1; C86853; AI256817; BF642806; 2600016F06Rik	
NM_009829	Vin1; Vin-1; C86853; AI256817; BF642806; 2600016F06Rik	
AV337593		
BB151515	p75; LNGFR; p75NTR; p75NGFR; Tnfrsf16	
NM_008005	FGF-18; D130055P09Rik	
AW986632	p96; Doc2; Doc-2; AA960054; AI957090; D15Wsu122e; 5730435J12Rik; Dab2	
BI151406	p75; LNGFR; p75NTR; p75NGFR; Tnfrsf16	
NM_008958	ptc2	
NM_013690	Hyk; Tie2; Cd202b	
NM_033597	c-myb; M16449; AI550390; MGC18531; Myb	
NM_007685	cryptic; AV265756	
AK008017	ngn3; Atoh5; Math4B; MGC129292; MGC129293	
BG071079	Ptc; mes; Ptc1; Ptch; A230106A15Rik	
NM_033597	c-myb; M16449; AI550390; MGC18531; Myb	
BC011513	c-myb; M16449; AI550390; MGC18531; Myb	
NM_023118	p96; Doc2; Doc-2; AA960054; AI957090; D15Wsu122e; 5730435J12Rik; Dab2	
BB622036	B-50; Basp2; GAP-43	
NM_009480	Usf1	
BB168690	Efs	
NM_007631	Cyl-1; PRAD1; bcl-1; AI327039	
NM_007631	Cyl-1; PRAD1; bcl-1; AI327039	
NM_007631	Cyl-1; PRAD1; bcl-1; AI327039	
AB042155	Opr; 1700049L20Rik	
D83147	Six2	
AA408768	D17860; AA408768; AI787086; MGC107649; prealbumin	
BC011272	Dey; Sey; Pax-6; 1500038E17Rik	
BM933729	haprin; D18Wsu100e	
BF687395	LKR; LOR; SDH; Lorsdh; LKR/SDH; LOR/SDH	
BQ174209	DRP2; Crmp2; Ulip2; TOAD-64; AI851130; Musunc33	
BC010291	Fgls; IP15; Cd225; mil-1; Cdw217; 1110004C05Rik	
NM_008885	Tr; HNPP; 22kDa; Gas-3; trembler	


Cluster b

Accession number	Gene Name	
W29605	Wnt-7b	
NM_010449	ERA1; Hox-1.6	
BG094302	AU024132; AU043124; Galpha13	
NM_009627	AM	
AV147875	MGC102095	
AV147875	MGC102095	
BC010197	CPH; fat; Cph1; Cph-1; R74677; MGC7101	
AV026617	c-fos; D12Rfj1	

HP1γ
BC018354	M32; HP1g; MGC118084; LOC382769; LOC544944	
Cluster d

Accession number	Gene Name	
BC006728	Myc2; Nird; Niard; c-myc; AU016757	
NM_009829	Vin1; Vin-1; C86853; AI256817; BF642806; 2600016F06Rik	
AV310588	Vin1; Vin-1; C86853; AI256817; BF642806; 2600016F06Rik	
AK007904	Vin1; Vin-1; C86853; AI256817; BF642806; 2600016F06Rik	
BQ175880	Vin1; Vin-1; C86853; AI256817; BF642806; 2600016F06Rik	
NM_009829	Vin1; Vin-1; C86853; AI256817; BF642806; 2600016F06Rik	
NM_009829	Vin1; Vin-1; C86853; AI256817; BF642806; 2600016F06Rik	
NM_009829	Vin1; Vin-1; C86853; AI256817; BF642806; 2600016F06Rik	
AV337593		
BB151515	p75; LNGFR; p75NTR; p75NGFR; Tnfrsf16	
NM_008005	FGF-18; D130055P09Rik	
AW986632	p96; Doc2; Doc-2; AA960054; AI957090; D15Wsu122e; 5730435J12Rik; Dab2	
BI151406	p75; LNGFR; p75NTR; p75NGFR; Tnfrsf16	
NM_008958	ptc2	
NM_013690	Hyk; Tie2; Cd202b	
NM_033597	c-myb; M16449; AI550390; MGC18531; Myb	
NM_007685	cryptic; AV265756	
AK008017	ngn3; Atoh5; Math4B; MGC129292; MGC129293	
BG071079	Ptc; mes; Ptc1; Ptch; A230106A15Rik	
NM_033597	c-myb; M16449; AI550390; MGC18531; Myb	
BC011513	c-myb; M16449; AI550390; MGC18531; Myb	
NM_023118	p96; Doc2; Doc-2; AA960054; AI957090; D15Wsu122e; 5730435J12Rik; Dab2	
BB622036	B-50; Basp2; GAP-43	
NM_009480	Usf1	
BB168690	Efs	
NM_007631	Cyl-1; PRAD1; bcl-1; AI327039	
NM_007631	Cyl-1; PRAD1; bcl-1; AI327039	
NM_007631	Cyl-1; PRAD1; bcl-1; AI327039	
AB042155	Opr; 1700049L20Rik	
D83147	Six2	
AA408768	D17860; AA408768; AI787086; MGC107649; prealbumin	
BC011272	Dey; Sey; Pax-6; 1500038E17Rik	
BM933729	haprin; D18Wsu100e	
		
Cluster e		
Accession number	Gene Name	
BF687395	LKR; LOR; SDH; Lorsdh; LKR/SDH; LOR/SDH	
BQ174209	DRP2; Crmp2; Ulip2; TOAD-64; AI851130; Musunc33	
BC010291	Fgls; IP15; Cd225; mil-1; Cdw217; 1110004C05Rik	
NM_008885	Tr; HNPP; 22kDa; Gas-3; trembler	
		
		
		
		
		
		
		
		
		
		
		
		
		
		
		
		
		
		
		
		
		
		
		
		
		
		
		
		
		
		
		
		
		
		
		
		
		
		
		
		
		
		
		
		
		
		
		
		
		
		
		
		
		
		
		
		
		
		
		
		
		
		
		
		
		
		
		
		
		
		
		
		
		
		
		
		
		
		
		
		
		
		
		
		
		
		
		
		
		
		
		
		
		
		
		
		
		
		
		
		
		
		
		
		
		
		
		
		
		
		
		
		
		
		
		
		
		
		
		
		
		
		
		
		
		
		
		
		
		
		
		
		
		
		
		
		
		
		
		
		
		
		
		
		
		
		
		
		
		
		
		
		
		
		
		
		
		
		
		
		
		
		
		
		
		
		
		
		
		
		
		
		
		
		
		
		
		
		
		
		
		
		
		
		
		
		
		
		
		
		
		
		
		
		
		
		
		
		
		
		
		
		
		
		
		
		
		
		
		
		
		
		
		
		
		
		
		
		
		
		
		
		
		
		
		
		
		
		
		
		
		
		
		
		
		
		
		
		
		
		
		
		
		
		
		
		
		
		
		
		
		
		
		
		
		
		
		
		
		
		
		
		
		
		
		
		
		
		
		
		
		
		
		
		
		
		
		
		
		
		
		
		
		
		
		
		
		
		
		
		
		
		
		
		
		
		
		
